# Gender impact on the outcome of rehabilitation programs in psychiatry: Brief report from a metropolitan residential rehabilitative service

**DOI:** 10.3389/fpsyt.2023.1145940

**Published:** 2023-03-30

**Authors:** Rita Cafaro, Chiara Ada Maria Rosti, Lucia Cerolini, Alberto Varinelli, Socrates Charitos, Roberta Magnotti, Beatrice Benatti, Bernardo Dell’Osso, Caterina A. Viganò

**Affiliations:** ^1^Department of Biomedical and Clinical Sciences “Luigi Sacco”, University of Milan, Milan, Italy; ^2^Department of Mental Health and Addiction, ASST Fatebenefratelli-Sacco, Milan, Italy; ^3^Department of Psychiatry and Behavioral Sciences, Stanford University, Stanford, CA, United States; ^4^Department of Health Sciences, "Aldo Ravelli" Center for Neurotechnology and Brain Therapeutic, University of Milan, Milan, Italy

**Keywords:** women, gender impact, psychiatric rehabilitation, rehabilitation programs, rehabilitation outcomes

## Abstract

**Background:**

Differences based on gender in the presentation and outcome of many psychiatric conditions have been highlighted in the past years. Moreover, women are often underrepresented in research samples, thus leading to a poorer understanding and addressing of their needs. As regards psychiatric rehabilitation, few studies have focused on the influence of gender on the outcomes of rehabilitation programs.

**Objectives:**

This study aimed to analyze the impact of gender on socio-demographic and clinical characteristics, as well as on main rehabilitation outcomes, in a sample of subjects undergoing rehabilitation programs in a metropolitan residential service.

**Methods:**

We collected socio-demographic, clinical variables and rehabilitation outcomes of all subjects discharged from the metropolitan residential rehabilitative service of the Luigi Sacco Hospital in Milan, Italy, from January 2015 to December 2021. Gender differences were analyzed through *t*-test and chi-square for continuous and categorical variables, respectively.

**Results:**

In a total sample of 129 subjects equally distributed for gender (50.4% women), all subjects improved after their rehabilitation program, as measured through specific psychometric scales. However, women had a higher proportion of discharges to their own household (52.3% vs. 25% of men). They also showed higher educational status (53.8% completed high school vs. 31.3% of men). Clinically, they showed longer duration of untreated illness (3.6 ± 7.31 vs. 1.06 ± 2.35 years) and lower frequency of substance use disorders compared to men (6.4% vs. 35.9%).

**Conclusion:**

The main result of this study shows, in light of an equal improvement in psychopathological and psychosocial functioning after the rehabilitation program, better outcomes in women compared to men, with a higher frequency of return to their own household after the completion of a rehabilitation program compared to men.

## Introduction

It is well known how many diseases can present differently between women and men. Nonetheless, gender-specific research that could aid to better identify and treat many conditions still represents a small portion of literature. Moreover, understanding gender differences can support the individualization of care, which is gaining huge importance in modern medicine. As regards psychiatric disorders, gender has been shown to influence prevalence, presentation, course of disease, comorbidities and response to treatment of many diseases (1).

However, many social, economic and cultural differences between men and women can influence and be influenced by clinical presentation, response to treatment and recovery (2). As an example, among patients with severe mental illnesses, women are more likely to be parents and have wider social and relational networks (3). The onset of psychiatric symptoms in major psychiatric diseases, especially psychotic syndromes, is usually 4–6 years later in women (4, 5); this difference might be related to the finding that women usually show better baseline levels of functioning, better personal care skills and greater involvement in occupational and social activities (6–8).

Focusing on these differences is crucial when considering that, for psychiatric disorders, remission of clinical symptoms alone is rarely associated with recovery (9, 10). The great impact of psychiatric conditions on psychosocial functioning can lead to long-term disability if not properly addressed during treatment. Moreover, functional and psychiatric disability can interest psychiatric patients regardless of their psychopathologic status (11).

The concept of recovery has been widely discussed in the psychiatric field and is becoming the main target of psychiatric care in many healthcare models (12–15). However, standardized models of care to facilitate recovery for the main psychiatric diseases are still debated (16, 17).

Mental health rehabilitation services focus on those with more severe and complex mental health problems and have been pioneers in adopting recovery based practices (18–20). Psychiatric rehabilitation can be defined as the set of interventions aiming to identify, reduce and prevent the causes of psychiatric disability, helping subjects to develop and use their resources and their personal skills in order to reach their goals and counteract the risk of chronic psychiatric illness (21–23).

Many studies focused on the efficacy of psychiatric rehabilitation in aiding patients to gain functional recovery (24–27). In the last decades, new models of psychosocial rehabilitation in individuals with long-term mental illnesses have been developed, mainly identified in evidence-based interventions focused on the individualization of rehabilitative programs (28–30); moreover, the implementation of outpatients and community-based services has become a priority in light of a multidimensional biopsychosocial approach to mental illness (31–35).

However, one of the main challenges in the development of optimal rehabilitation models, is the difficulty in identifying and standardizing outcome measures of rehabilitative programs; objective measures of improvement in psychopathological, relational and social functioning are needed (10, 36–38). Few studies focused on the identification of efficient outcome measures. In a recent research, Vanzetto and colleagues identified (i) improvement in validated psychometric scales, (ii) reduction of hospitalizations, (iii) improved continuity of care, (iv) adherence to a long acting injectable (LAI) antipsychotic, and (v) a stable employment for at least 1 year after discharge as reliable outcome measures of improved global functioning (39). Another outcome measure often used in literature is the destination at discharge from the rehabilitation program, an indirect measure of global functioning that identifies if a patient can return to an independent living environment or needs further care.

Moreover, few studies focused on the identification of factors influencing rehabilitation outcomes, and even less focused on the influence of gender on rehabilitation outcomes. In one study, Cook analyzed data from 650 subjects who carried on rehabilitation programs in a psychosocial rehabilitation center in the US (26). Considering the destination at discharge as major outcome measure, he found five factors associated with returning to an independent living environment: reaching higher levels of functioning, having longer rehabilitation programs, not receiving continuous support from psychiatric services, being parents and participating more in social and occupational activities during rehabilitation. He found that women were more likely to be parents and to participate in social and occupational activities, being therefore more likely to return to their own household after a rehabilitation program.

In light of the paucity of data on the influence of gender on rehabilitation outcomes in psychiatry, we aimed to study gender differences in a sample of subjects undergoing community-based rehabilitation programs in Milan, focusing on the possible different outcomes between the two genders.

## Materials and methods

This retrospective observational study aimed to evaluate the impact of gender on socio-demographic and clinical characteristics and on rehabilitation outcomes of residential patients carrying out rehabilitative programs at the High Assistance Rehabilitation Community (HARC) of the “Luigi Sacco” University Hospital, belonging to the Department of Mental Health and Addiction of the ASST Fatebenefratelli – Sacco, Milan, Italy. The HARC provides continuous residential assistance to patients with major psychiatric disorders who voluntarily accept to undergo a rehabilitation program. The study protocol was approved by the Department of Psychiatry of the ASST Fatebenefratelli-Sacco of Milan as relevant institutional review board for low-risk studies.

All patients must be previously taken in charge at a territory-based psychosocial outpatients service responsible for continuity of care, which is also responsible for requesting admissions to HARC. Patients can undergo two different residential rehabilitative programs: the Post-Acute (RPA), 3 months program, renewable up to 6 months, and the High Intensity (RHI), 18 months program, extendable up to 24 months.

Patients who are admitted to the HARC undergo a psychopathological and psychosocial functioning evaluation before identifying and planning, in accordance with the patient preferences, individualized goals within the personalized therapeutic rehabilitation pathway. Activities involved in rehabilitation programs are individual and group occupational projects, psycho-educational interventions, cognitive remediation programs, social skills training, expressive and psychotherapeutic activities, all of which aiming to develop and improve personal, social, relational and work-related skills. Every patient is offered an individualized program of activities tailored to their rehabilitation needs, informed by their psychosocial and functional assessment.

The evaluation of psychopathological status and psychosocial functioning is made through specific scales: Kennedy Axis V (40, 41), from which the score of the Global Assessment of Functioning Scale (GAF) (42) is also derived, Brief Psychiatric Rating Scale (BPRS) (43), Life Skills Profile (LSP) (44, 45) and the AR (Aree Riabilitative – Rehabilitation Areas) module of the VADO (Valutazione di Abilità, Definizione di Obiettivi – Skills Evaluation, Goals Definition) scale (46). These scales are used first as an indicator of baseline functioning of each patient, and secondly, when repeated at the end of the rehabilitation program, as rehabilitation outcome measures.

Medical records of all residential HARC patients discharged between January 2015 and December 2021 were retrospectively reviewed, in accordance with hospital privacy rules, by authorized personnel in order to collect pseudoanonymized, clinical, socio-demographic and therapeutic data. Collected variables were: gender (used to stratify the sample), age at admission, educational status, housing and work condition at admission, psychiatric diagnosis, presence of dual diagnosis, organic comorbidities (i.e., medical conditions requiring long term treatment such as hypothyroidism, diabetes, hypertension), age at onset and at first psychiatric treatment, duration of illness and duration of untreated illness, previous rehabilitation experiences both lifetime and in the 12 months before admission, number of hospitalizations lifetime, place of origin at the time of admission (Psychiatric ward, Own household, Other community, Day hospital), reason of admission, type of rehabilitation program (RPA vs. RHI), length of rehabilitation program (months), hospitalizations during the project, destination after the program, scores of the previously mentioned scales at both admission (T0) and discharge (T1).

The change in the scores between T0 and T1 (i.e., improvement vs. worsening) and the destination of the patient after the program (i.e., his own household, other rehabilitative community, protected housing, hospitalization, drop-out) are considered outcome measures of the rehabilitation program. Dropouts have been identified in both those patients who voluntarily interrupted the rehabilitation program before its end, and in those who have been discharged beforehand because of non-compliance to the rules of the community.

### Statistical analyses

Categorical variables are presented using frequencies and percentages, while continuous variables are presented as mean and standard deviation (SD). Gender has been used to stratify the sample and compare variables between subjects. Chi-square test with Bonferroni post-hoc analysis and Student *t*-test have been used for categorical and continuous variables, respectively. A paired-samples *t*-test has been carried out to analyze the change in the scores between T0 and T1, in the total sample and in the two subsamples based on gender. Statistical analyses were performed with SPSS software, version 26. Statistical significance has been set at value of *p* < 0.05 for all analyses.

## Results

The final sample included 129 subjects discharged from the HARC between January 2015 and December 2021. The distribution between genders was almost 1:1 [65 (50.4%) women, 64 (49.6%) men]. Socio-demographic and clinical characteristics of subjects in the total sample and their comparison between genders are shown in Table 1.

**Table 1:** Socio-demographic and clinical characteristics in the whole sample and their comparison based on gender.

	Total sample 129 (100%)	Women 65 (50.4%)	Men 64 (49.6%)
**Age at admission (years)**	38.98 ± 14.06	40.00 ± 13.05	37.95 ± 15.04
**Educational status**			
Primary school	7 (5.4%)	4 (6.2%)	3 (4.7%)
Secondary school	56 (43.4%)	24 (36.9%)	32 (50%)
High school	55 (42.6%)	35 (53.8%)*	**20 (31.3%)**
University	6 (4.7%)	1 (1.5%)	5 (7.8%)
**Employment status**			
Employed	20 (15.5%)	12 (18.5%)	8 (12.5%)
Unemployed	107 (82.9%)	52 (80.8%)	55 (85.9%)
**Housing condition**			
Alone	42 (32.5%)	26 (40%)	16 (25%)
Family	70 (54.3%)	34 (52.3%)	36 (56.3%)
Community	7 (5.4%)	1 (1.5%)	6 (9.3%)
Protected housing	5 (3.9%)	2 (3.1%)	3 (4.7%)
**Age at onset (years)**	21.06 ± 9.70	20.66 ± 9.08	21.47 ± 10.34
**Age at first psychiatric treatment (years)**	23.77 ± 11.52	25.18 ± 11.29	22.33 ± 11.66
**Duration of illness (years)**	13.53 ± 12.32	14.82 ± 12.03	12.23 ± 12.56
**DUI (years)**	2.34 ± 5.58	3.60 ± 7.31*	**1.06 ± 2.35**
**Psychiatric diagnosis**			
Psychotic disorder	50 (38.7%)	20 (30.8%)	30 (46.9%)
Bipolar disorder	29 (22.5%)	16 (24.6%)	13 (20.3%)
Personality disorder	17 (13.2%)	11 (16.9%)	6 (9.4%)
Schizoaffective disorder	13 (10.1%)	9 (13.9%)	4 (6.2%)
MDD	10 (7.8%)	6 (9.2%)	4 (6.2%)
OCD	7 (5.4%)	1 (1.5%)	6 (9.4%)
Anxiety disorder	3 (2.3%)	2 (3.1%)	1 (1.6%)
**Dual diagnosis**			
SUD	27 (20.9%)	**4 (6.2%)**	23 (35.9%)*
Intellectual disability	10 (7.8%)	5 (7.7%)	5 (7.8%)
SUD + intellectual disab.	2 (1.6%)	2 (3.1%)	0
Gambling	1 (0.8%)	1 (1.5%)	0
**Organic comorbidities**			
No comorbidities	61 (47.4%)	27 (41.6%)	34 (53.1%)
1 comorbidity	19 (14.7%)	9 (13.8%)	10 (15.6%)
>1 comorbidity	49 (37.9%)	29 (44.6%)	20 (31.3%)
**Previous rehabilitation**			
Lifetime	81 (62.8%)	39 (60.9%)	42 (64.6%)
12 months	61 (47.3%)	35 (53.5%)	26 (40.6%)
**Hospitalizations lifetime**			
0–1	19 (14.7%)	9 (13.8%)	10 (15.6%)
2–4	39 (30.2%)	16 (24.6%)	23 (35.9%)
5–9	26 (20.2%)	15 (23.1%)	11 (17.2%)
≥ 10	40 (31%)	24 (36.9%)	16 (25%)
**Origin at admission**			
Psychiatric ward	83 (64.3%)	45 (69.2%)	38 (59.4%)
Own household	37 (28.7%)	15 (23.1%)	22 (34.4%)
Other community	2 (1.6%)	1 (1.5%)	1 (1.6%)
Day hospital	3 (2.3%)	2 (3.1%)	1 (1.6%)
**Reason of admission**			
Clinical stabilization	92 (71.3%)	49 (75.4%)	43 (67.2%)
Recovery of social skills	19 (14.7%)	9 (13.8%)	10 (15.6%)
Living environment issues	13 (10.1%)	5 (7.7%)	8 (12.5%)
**Rehabilitation program**			
RPA	63 (48.8%)	35 (53.8%)	28 (43.7%)
RHI	66 (51.2%)	30 (46.2%)	36 (56.3%)
**Duration of program (months)**			
≤3 months	56 (43.4%)	29 (44.6%)	27 (42.2%)
4–6 months	22 (17.1%)	11 (16.9%)	11 (17.2%)
7–12 months	27 (20.9%)	14 (21.5%)	13 (20.3%)
13–18 months	10 (7.8%)	5 (7.7%)	5 (7.8%)
≥18 months	14 (10.9%)	6 (9.2%)	8 (12.5%)
**Hospitalizations during the program**			
No	108 (83.8%)	54 (83.1%)	54 (84.4%)
1–2	18 (13.9%)	10 (15.4%)	8 (12.5%)
>2	3 (2.3%)	1 (1.5%)	2 (3.1%)

DUI, duration of untreated illness; MDD, major depressive disorder; OCD, obsessive–compulsive disorder; SUD, substance use disorder; RPA, Post-acute rehabilitation; RHI, High intensity rehabilitation. *p < 0.05.

For the majority of socio-demographic variables, women and men showed similar distribution. However, a significantly greater proportion of women reached a higher educational status before admission, with 35 (53.8% of women) of them having a high school diploma, compared with 20 (31.3% of men) men (Chi-square 9.836, *p* < 0.05).

As regards clinical characteristics, women showed a slightly longer duration of untreated illness (3.6 ± 7.31 vs. 1.06 ± 2.35 years, *t* 2.644, *p* < 0.05), while between men a significantly greater proportion of subjects presented a dual diagnosis, with 23 (35.9% of men) of them presenting a co-occurring substance use disorder diagnosis compared with 4 (6.2% of women) women.

All the rehabilitative features of the projects carried on by the subjects included in this study are comparable between men and women. Comparable proportions of subjects of the two genders were enrolled in high intensity programs and post-acute programs, and the duration of programs was similar in the two genders. Origin at admission and reason of admission were comparable in the two subsamples as well.

**Figure 1:**
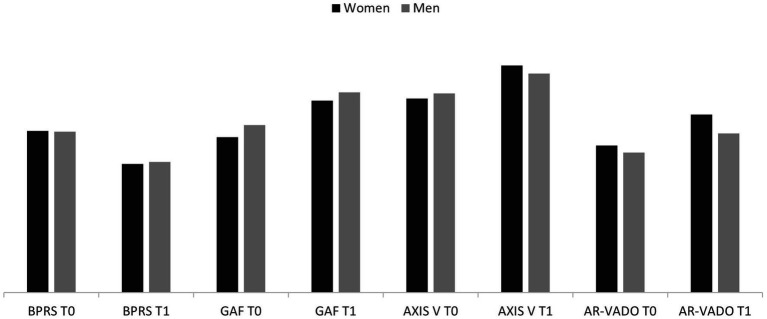
Psychometric scales scores at T0 and T1 and their comparison based on gender BPRS, Brief Psychiatric Rating Scale; GAF, Global Assessment of Functioning; Axis V, Kennedy axis V; AR-VADO, Aree Riabilitative – Rehabilitation Areas – module of the VADO (Valutazione di Abilità, Definizione di Obiettivi – Skills Evaluation, Goals Definition) scale.

In terms of rehabilitation outcome measures, identified both as a change in the scores of Kennedy Axis V, BPRS, AR-VADO and GAF scales between T0 and T1 and in the destination at discharge after a rehabilitation program (Table 2), no difference between women and men was found in the baseline scores, therefore the two subsamples could be considered comparable. As regards T1, we found an overall statistically significant improvement in all subjects, regardless of gender (Figure 1). When comparing destinations at discharge between women and men, we found a greater proportion of women discharged to their own household after the rehabilitation project, compared to a smaller proportion of men [34 (52.3%) women vs. 16 (25%) men, chi-square 13.214, *p* < 0.01]. Moreover, even though not reaching a statistical significance, it is worth noting how a greater proportion of dropouts was found in the men’s subsample compared to women [17 (26.2%) women vs. 26 (40.6%) men].

**Table 2:** Outcome measures in the whole sample and their comparison based on gender.

	Total sample	Women	Men
**Destination at discharge**			
Own household	50 (38.8%)	34 (52.3%)*	**16 (25%)**
Other community	22 (17.1%)	11 (16.9%)	11 (17.2%)
Protected housing	7 (5.4%)	1 (1.5%)	6 (9.4%)
Psychiatric ward	7 (5.4%)	2 (3.1%)	5 (7.8%)
Dropouts	43 (33.3%)	17 (26.2%)	26 (40.6%)
**BPRS**			
T0	44.6 ± 10.9	44.7 ± 10.8	44.5 ± 11.0
T1	35.8 ± 11.2	35.5 ± 11.9	36.1 ± 10.5
GAF			
T0	44.6 ± 11.6	42.9 ± 11.7	46.3 ± 11.4
T1	54.1 ± 14.4	53.0 ± 16.1	55.3 ± 12.6
**Kennedy Axis V**			
T0	54.3 ± 10.5	53.6 ± 10.4	55.1 ± 10.5
T1	61.6 ± 12.2	62.7 ± 13.8	60.5 ± 10.5
**AR-VADO**			
T0	39.6 ± 12.5	40.6 ± 12.8	38.7 ± 12.4
T1	46.7 ± 15.3	49.2 ± 15.2	44.0 ± 15.1

BPRS, Brief Psychiatric Rating Scale; GAF, Global Assessment of Functioning; AR-VADO, Aree Riabilitative – Rehabilitation Areas – module of the VADO (Valutazione di Abilità, Definizione di Obiettivi – Skills Evaluation, Goals Definition) scale. *p < 0.05.

## Discussion

The aim of this study was to determine whether gender might influence the outcomes of psychiatric rehabilitation, analyzing its relationship with main socio-demographic, clinical and rehabilitative characteristics in a sample of subjects who carried on rehabilitation programs at the HARC, a high intensity and post-acute rehabilitation community in the metropolitan area of Milan.

One outcome measure analyzed consisted in a change of main psychopathological and functional scales scores from T0 to T1. In our sample, we found a statistically significant improvement in all scales at T1, regardless of gender. In a general perspective, this result confirms the efficacy of the individualized rehabilitation programs carried on at the HARC. Of note, no difference was found in the T0 scores of psychosocial and psychopathologic scales of female and male subjects, therefore the two subsamples could be considered comparable,

The second outcome measure consisted in the destination at discharge from the community. Destination at discharge is influenced by various factors, mainly the level of independence achieved during the program, the successful stabilization of symptoms and the acquisition or improvement of social, relational and work-related skills (26). The importance of work-related skills, moreover, has been recognized in recent years (47). In our sample, we found that a greater proportion of women was discharged to their own household, therefore reflecting a higher level of functioning reached during the rehabilitation program.

Many studies in the literature confirm the similar levels of improvement in functioning between the two genders, as measured by psychometric scales, regardless of destination at discharge (26, 48). In this perspective, environmental factors could influence the destination at discharge, regardless of the level of functioning of subjects. Family and economic issues, for example, might influence the possibility of returning to one’s own household.

Even though the higher number of women discharged to their own household is in line with previous findings (26), we did not identify in our sample specific reasons for this difference, beside the possibility that a different mean educational status might reflect a different level of baseline functioning for men and women. In fact, it is needed to acknowledge that T0 scores of psychometric scales is collected at admission to the community, and therefore usually after an acute episode.

Moreover, many studies focused on the higher probability for women to develop social skills and build social and sentimental relationships before the onset of a psychiatric disorder, especially in the psychotic spectrum (8, 48–50). In our sample, however, it was not possible to identify and analyze specific social skills. Moreover, even though not reaching statistical significance, in our sample more men had a psychotic spectrum disorder compared to women. This data might relate to levels of functioning found in women and men of our rehabilitation service.

Another reason for this difference could lay in the higher frequency of dual diagnosis found in the male subsample, which could have influenced both functioning and the possibility to return to one’s own household. The higher number of men reporting a substance use disorder is in line with previous findings (48, 50, 51).

Lastly, in our sample women showed a longer duration of untreated illness compared to men. This result is in contrast with previous data, that underline how women usually refer to psychiatric services earlier and with higher adherence compared to men (52). Such finding might be explained in light of the higher proportion in our sample of women diagnosed with psychiatric disorders usually presenting with a longer duration of untreated illness compared to men (i.e., more women were diagnosed with major depressive disorder, anxiety disorder and obsessive–compulsive disorder). Moreover, the long duration of untreated illness found in the female subsample of this study, might have had a role in reducing baseline levels of psychopathological and psychosocial functioning.

In our study, we aimed to analyze potential gender differences in the outcomes of rehabilitation programs of a rehabilitative community in Milan. Our main result showed how more women are discharged to their own household after a rehabilitation program, compared to men. This result is in line with previous literature, and might reflect a higher level of baseline functioning in women, which usually develop more social and relational skills before the onset of the disease. However, through the use of validated psychometric scales for the evaluation of psychosocial and psychopathological functioning both at admission and discharge, no major difference between men and women was found, and an overall improvement in functioning characterized the sample.

This study has several strengths, such as the relatively large sample of subjects included and the thorough submission of validated psychometric scales to all subjects. However, one limitation of the study lays in the lack of reliable data about patients’ baseline levels of functioning, i.e., before the acute episode or condition that required admission to the rehabilitative community. In fact, our results and previous findings in literature suggest different levels of functioning in female and male subjects with psychiatric conditions. Moreover, influence of previous traumatic events (i.e., violence or abuse) on psychopathological functioning has not been included in the present research, in order to keep the focus on gender alone. However, we must acknowledge the greater frequency of such events in the female gender, and therefore the importance of including these data in future studies. Lastly, our research did not focus on characterizing subjects who showed worse rehabilitation outcomes, therefore no hypothesis on which specific features might be more directly related to improvement during rehabilitation can be drawn from our study. Further research focusing on gender-specific and rehabilitation psychiatry is needed, in order to aid mental health services to implement preventive and supportive activities for the acquisition and improvement of specific skills associated with higher probability of independent living.

## Data availability statement

The raw data supporting the conclusions of this article will be made available by the authors, without undue reservation.

## Author contributions

All authors listed have made a substantial, direct, and intellectual contribution to the work and approved it for publication.

## Funding

The authors acknowledge support from the University of Milan through the APC initiative.

## Conflict of interest

The authors declare that the research was conducted in the absence of any commercial or financial relationships that could be construed as a potential conflict of interest.

## Publisher’s note

All claims expressed in this article are solely those of the authors and do not necessarily represent those of their affiliated organizations, or those of the publisher, the editors and the reviewers. Any product that may be evaluated in this article, or claim that may be made by its manufacturer, is not guaranteed or endorsed by the publisher.
